# TSOL18 vaccine and oxfendazole for control of *Taenia solium* cysticercosis in pigs: A field trial in endemic areas of Tanzania

**DOI:** 10.1371/journal.pntd.0008785

**Published:** 2020-10-14

**Authors:** Mwemezi L. Kabululu, Helena A. Ngowi, James E. D. Mlangwa, Ernatus M. Mkupasi, Uffe C. Braae, Angela Colston, Claudia Cordel, Elizabeth J. Poole, Kristin Stuke, Maria V. Johansen

**Affiliations:** 1 Tanzania Livestock Research Institute (TALIRI)—Uyole, Mbeya, Tanzania; 2 Department of Veterinary Medicine and Public Health, College of Veterinary Medicine and Biomedical Sciences, Sokoine University of Agriculture, Morogoro, Tanzania; 3 Department of Infectious Disease Epidemiology, Statens Serum Institut, Copenhagen, Denmark; 4 One Health Center for Zoonoses and Tropical Veterinary Medicine, Ross University School of Veterinary Medicine, Basseterre, Saint Kitts and Nevis; 5 Global Alliance for Livestock Veterinary Medicines (GALVmed), Nairobi, Kenya; 6 Global Alliance for Livestock Veterinary Medicines (GALVmed), Bloemfontein, Free State, South Africa; 7 Statistics for Sustainable Development (Stats4SD), Reading, United Kingdom; 8 Section for Parasitology and Aquatic Pathobiology, Department of Veterinary and Animal Sciences, Faculty of Health and Medical Sciences, University of Copenhagen, Frederiksberg, Denmark; Universidad Nacional Autónoma de México, MEXICO

## Abstract

A field trial was conducted in Tanzania to determine the effectiveness of TSOL18 vaccine used concurrently with oxfendazole (OFZ), and of OFZ alone, on *T*. *solium* cysticercosis determined by organ and half carcase dissection of slaughter age pigs. This study followed a quasi-experimental group design. Suitable trial sites were randomly allocated to either treatment group T1 (OFZ treatment alone [30mg/kg, Paranthic 10%]) or T2 (TSOL18 [1ml, Cysvax] plus OFZ). Three 4-monthly treatments were administered to eligible pigs. A random selection of pigs were necropsied at baseline and at endline, 2–3.5 months after the final treatment. Additionally, untreated pigs from T1 and T2 areas were necropsied at endline to provide contemporaneous comparisons with T1 and T2 pigs. Baseline prevalence of viable *T*. *solium* cysticerci for T1 was 25.5% (Exact 95% CI: 13.9, 40.3; n = 12/47), and for T2 was 12.0% (CI: 6.4, 20.0; n = 12/100). At endline, prevalence was 2.8% for T1 (CI: 0.1, 14.5, n = 1/36) and 0% for T2 (CI: 0, 4.7, n = 0/77). Among untreated pigs, three had viable cysticerci, one from T1 area (12.5%, CI: 0.3, 52.7; n = 1/8) and two from T2 area (5.7%, CI: 0.7, 19.2, n = 2/35). Fisher’s exact test showed significant changes in prevalence from baseline to endline in both groups (T1: *p =* 0.005, T2: *p =* 0.001). Firth’s penalized Maximum Likelihood method suggested the changes were not significant relative to their controls (T1: *p =* 0.245, T2: *p =* 0.076). These findings showed a significant reduction in the prevalence of viable cysticerci from baseline to endline after both interventions. However, the changes could not be definitively attributed to the interventions due, in part, to small numbers of control pigs. Concurrent administration of the TSOL18 and OFZ cleared infection among assessed pigs whereas infection remained after treatment with OFZ only. Further studies including larger sample sizes would be required for more definitive conclusions. A One Health approach is recommended for rapid and sustainable impact.

## Author summary

The pork tapeworm *T*. *solium* affects the health and income of poor people in endemic areas. A pig vaccine—TSOL18, and a drug—oxfendazole (OFZ) present options for its control. In this study we assessed the effectiveness of the two tools against the parasite in pigs. We provided one group of pigs (T1) with a concurrent administration of TSOL18 and OFZ, and another group (T2) with OFZ only, in three rounds at 4 months intervals. We compared infection prevalence before and after the interventions by detection of cysticerci through carcase dissections. In addition, untreated pigs from the T1 and T2 areas were also sacrificed to serve as controls. We found a significant reduction in prevalence within the groups after the interventions. However, the reductions were not significant when compared with the reductions in the control groups, hence could not be definitively attributed to the interventions. Further, concurrent administration of TSOL18 and OFZ cleared infection among assessed pigs whereas OFZ alone did not clear the infection. Further studies would be required to make more definitive conclusions. We advocate for a One Health approach for a more rapid and sustainable impact.

## Introduction

*Taenia solium* is an important but neglected zoonotic tapeworm causing two distinct diseases, taeniosis in humans and cysticercosis in pigs and humans. The public health significance of the parasite arises when cysticerci lodge in the brain of humans, causing a potentially life-threatening condition—neurocysticercosis (NCC). NCC is identified as a major cause of deaths among foodborne diseases. On a global scale, *T*. *solium* was identified to have the highest public health importance amongst the foodborne parasites [1,2].

*T*. *solium* is prevalent in low- and middle-income countries in sub-Saharan Africa [3], Latin America [4] and South and southeast Asia [5]. In these areas, NCC is the major cause of preventable epilepsy in humans [6–8], and when access to health services is limited, mortality due to NCC is reported to be up to six times higher than in the general population [9]. Furthermore, porcine cysticercosis (PC) can cause serious economic impacts among smallholder pig farmers. The farmers might incur serious financial losses when their infected pigs cannot be sold, are sold at reduced prices, or are condemned without compensation upon slaughter [10,11].

Despite being declared eradicable by the International Task Force on Disease Eradication (ITFDE) in 1992 [12,13], *T*. *solium* remained neglected and a major economic and public health concern in endemic areas. In 2010, the World Health Organization (WHO) included *T*. *solium* infections in a list of Neglected Tropical Diseases (NTDs) as a way of increasing advocacy for controlling the disease [14]. However, while it appears that prevention and control of *T*. *solium* should be straightforward and practical, various intervention strategies seem to have largely failed to interupt transmission [15]. In Tanzania for example, improvement in pig confinement could not significantly reduce prevalence of *T*. *solium* cysticercosis indicating, among other things, the possibility of in-pen transmission [16]. In addition, three years of annual treatment of school-aged children with praziquantel led to a significant reduction in the prevalence of PC [17]. However, persistence of tapeworm carriers undermines the long-term impact of such intervention if used as the only intervention for elimination. Health education has been seen to improve knowledge, at least in the short-term [18–20] and reduce the incidence of PC [20]. However, under these settings, health education had no significant effect on risky practices which would result in the continued transmission of the disease.

TSOL18 vaccine for the prevention of *T*. *solium* infection in pigs and oxfendazole (OFZ) for killing cysticerci in pig tissues are relatively new tools and present an opportunity for effective and sustainable control of the disease in pigs. TSOL18 (150μg recombinant protein in mineral oil adjuvant) has been identified as the most efficacious vaccine currently available against PC by providing 100% protection against the disease [21,22]. OFZ, a benzimidazole, on the other hand, has been proven to be the most efficacious anthelmintic against *T*. *solium* cysticerci in pig muscles with no reported side effects but with limited effect on brain cysticerci. TSOL18 vaccination prevents new infections in vaccinated pigs but does not clear cysticerci that may have established in the tissues prior to vaccination. Hence, a concurrent use of the vaccine plus oxfendazole, would clear any pre-existing infection and prevent new infections, and therefore offers the best potential for clearing of infection in treated pigs. Therefore, it was anticipated that systematic, programmatic use of both products in the same population of animals would provide a very effective means to rapidly reduce the prevalence of infection in pigs.

This study assessed the effectiveness of concurrent use of TSOL18 (Cysvax, IIL, India) and OFZ (Paranthic 10%, MCI, Morocco), and of OFZ alone when applied at least two times at an interval of four months, on the prevalence and counts of viable *T*. *solium* cysticerci in slaughter age pigs exposed to natural infection in Mbeya Rural and Mbozi districts in the southern highlands of Tanzania.

## Materials and methods

### Study sites

The study was implemented from October 2016 to January 2018 in Mbeya Rural and Mbozi districts in Tanzania. The two districts had a total of 59 wards and 295 villages and human and pig populations were estimated to be 751,658 [23] and 43,865 (unofficial reports, District Livestock Offices), respectively. The districts are largely rural and the majority of people practice subsistence mixed farming including pig keeping. The districts were selected based on previous publications which indicated that *T*. *solium* was endemic in the districts [16,24,25]. Carcase dissections were done at a makeshift post-mortem facility at Tanzania Livestock Research Institute (TALIRI)-Uyole centre, in Mbeya municipality. Pig slaughter and carcase processing were done at the nearest public slaughter slab.

### Study design

This study was a quasi-experimental group design comparing pre-intervention (baseline) and post-intervention (endline) prevalence and counts of viable *T*. *solium* cysticerci within treatment groups T1 (OFZ only) and T2 (TSOL18 concurrently with OFZ); and between each group and its endline control. Selected trial sites were randomly allocated to receive either of the two interventions. Pigs for baseline and endline carcase dissections were randomly selected from populations of untreated and treated pigs, respectively. A control group comprised of untreated pigs from the same trial sites was added at endline carcase dissections to provide contemporaneous comparison with T1 and T2 groups. The baseline values for both T1 and T2 groups were also used as their baseline control values. Prevalence and counts of cysticerci were determined by meticulous organ and half carcase dissections of a random sample of slaughter-age pigs before and after the interventions.

### Selection of study units and randomization

**Fig 1:**
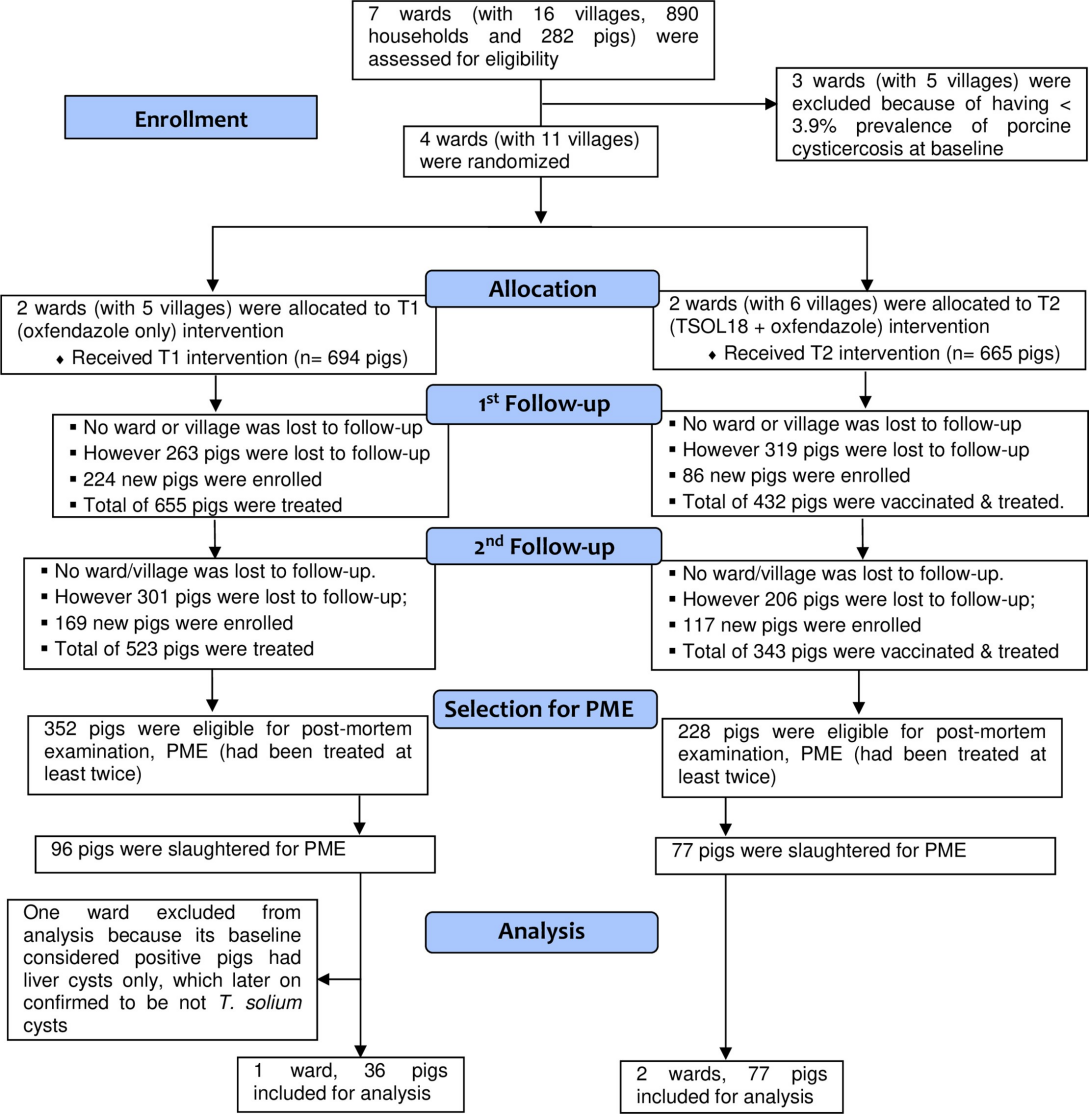
Flow of study units and activities during an intervention trial with TSOL18 and oxfendazole, Tanzania.

Seven wards, three in Mbeya Rural district and four in Mbozi district, were assessed for eligibility (Fig 1). Based on the results of the baseline carcase dissections, four wards (comprising 11 villages) with the highest prevalence were selected for the study; two in Mbeya Rural district (Mshewe and Iyunga Mapinduzi) and two in Mbozi district (Igamba and Magamba) (Table 1). Due to substantial differences in prevalence between the wards, they were paired, balanced on their prevalence, such that each ward-pair had a combined prevalence of ≥10%. Each ward-pair was comprised of a ward from each district, that is, Iyunga-Igamba (with a combined prevalence of 12%) and Magamba-Mshewe (prevalence of 14.3%). The ward-pairs were randomly allocated to T1 (Magamba-Mshewe) and T2 (Iyunga-Igamba). Mshewe ward was subsequently excluded from the study after its baseline prevalence was determined to be 0% following molecular confirmatory tests on suspicious cysticerci showed that none of the pigs from the ward had *T*. *solium* cysticerci apart from one pig that had only one non-viable cyst.

**Table 1:** Baseline prevalence of *Taenia solium* cysticercosis determined by organ and half carcase dissections of slaughter-age pigs in seven assessed wards in Mbeya Rural and Mbozi districts, southern highlands of Tanzania.

District	Ward	N	%	Treatment group
Mbozi	Magamba	47	25.5	T1
Mbozi	Igamba	50	16.0	T2
Mbeya	Iyunga Mapinduzi	50	8.0	T2
Mbeya	Mshewe^1^	51	3.9	T1
Mbozi	Mlangali	27	3.7	NS
Mbozi	Isansa	30	3.3	NS
Mbeya	Bonde la Songwe	27	0	NS

n, number of pigs examined; T1, intervention with oxfendazole only; T2, concurrent intervention with TSOL18 and oxfendazole; NS, not selected.

1 Mshewe ward was selected for the study based on a suspected prevalence of 3.9%. The ward was later excluded from the study after molecular confirmatory tests on suspicious cysticerci showed that none of the pigs had viable *T*. *solium* cysticerci.

In the selected sites, all pig farmers willing to participate in the study were included. In each household, all pigs that satisfied the inclusion criteria were enrolled for the intervention trial. These were at least eight weeks of age, with a bodyweight of at least 3 kg, apparently healthy, and neither within four weeks of farrowing nor within eight weeks of slaughter. Rolling or continual enrollment throughout the study was permitted such that after the initial enrolments, new households and pigs that met the inclusion criteria were enrolled at subsequent visits. Households were identified by the name of the household head and the Global Positioning System (GPS) coordinates. Enrolled pigs were identified by using duplicate unique numbered ear tags attached to both ears. Animal details were recorded including ear tag number, estimated age, sex, and confinement status of a pig.

For the baseline carcase dissections, pigs representative of the slaughter-age pig populations in study sites were selected from consenting owners by using computer-generated random numbers. In each selected household, one eligible pig was bought for slaughter. An eligible pig was at least six months of age and apparently healthy. For the endline carcase dissections, computer-generated random numbers were also used to select pigs from among those which had been treated at least twice. In addition, any available untreated pigs from the same trial sites, satisfying the same inclusion criteria as the pigs selected for the baseline carcase dissections, were also included for endline carcase dissection to provide the endline control groups.

### Sample size

Samples sizes were calculated at a 5% level of significance, 80% power, 1-sided test, odds ratios varying from 0.1–0.3. Initially, 55 pigs were required per group assuming an initial prevalence of 20% and anticipating an odds ratio of 0.2 and an endline prevalence of 4.8%. After the baseline prevalence was found to be <15% (14.3% for T1 and 12% for T2) sample size was recalculated to at least n = 96 per group. However, after one T1 ward was dropped leaving a T1 baseline prevalence of 25.5%, this would have required a sample size of only n = 36 for endline necropsies, lower than the original sample size (n = 55) which assumed 20% prevalence. The sample size for T2 was determined to be n = 90 using the observed baseline prevalence of 12%, the same odds ratio of 0.2 but requiring a reduction in prevalence to 2.65%. The sample size for the endline controls in each group were determined to be the same as the sample sizes for their respective treatment groups for endline necropsies, that is n = 36 for T1 and 90 for T2.

### Pig treatment and vaccination

Vaccinations and treatments were conducted in three rounds at intervals of four months, in the year 2017 between January and February (first round), May and June (second round) and September and October (third round). Each vaccination/treatment round was completed within four weeks in both treatment groups and across both districts. During vaccination/treatment, a pig was restrained in a standing position, its head stabilized by using a snare. Piglets were lifted and handled off the ground. Body weights were estimated to ascertain whether a pig was suitable for inclusion and to determine the correct OFZ dosage. TSOL18 vaccine was administered intramuscularly at a standard dose of 1 ml on the left side of the neck behind the base of the ear using a good aseptic technique. OFZ was administered orally as a single dose of 3 ml/kg body weight (30mg/kg body weight) by using a graded dosing syringe. A laminated body weight/dose chart was used to ensure accurate dose determination. Farmers were advised to observe the 21 days withholding period for OFZ.

### Carcase dissections

Baseline carcase dissections were done between November and December 2016 while endline carcase dissections were done between November 2017 and January 2018, about 10 ½ months after the first intervention round. Purchased pigs were transported to a holding facility at TALIRI-Uyole. Thereafter, they were taken to a nearby public slaughter slab and were slaughtered and processed as per the slaughter slab procedures. After opening the carcases, the heart and the diaphragm were separated and were labelled with the animal ID to match them with the respective carcases. The organs and the carcases were transported to the post-mortem facility at TALIRI-Uyole where the head was separated and the tongue, the brain and masticatory muscles were extracted. The carcases were divided cranio-caudally into two halves and only the right half of the carcase was used. The muscles of the half carcase were excised from bones, and were grouped into muscles of the forelimb and muscles of the remaining half carcase. All organs and extracted muscle groups were labelled with animal ID to ensure the traceability of all data.

The organs and muscle groups were dissected with sagittal cuts of approximately 0.3 cm in thickness. The cut surfaces were examined for presence, viability and number of *T*. *solium* cysticerci. A cysticercus was classified as viable if it was a translucent vesicle with a visible scolex inside. A non-viable cyst was one that was smaller, non-translucent, filled with dense whitish to yellowish fluid, or containing fibrous or caseous (calcified) material. A pig was considered to be infective if it had at least one viable *T*. *solium* cysticerci in the examined organs/muscles. In heavily infected carcases, the whole brain, tongue, masticatory muscles, heart and diaphragm were dissected, but for the carcase musculature, a representative sample weighing 1 kg was dissected and the number of cysticerci for the remaining muscle mass was estimated based on its weight. Total cysticerci count for a pig was estimated as double the number for half carcase musculature plus numbers for the brain, tongue, masticatory muscles, heart and diaphragm. Dissected carcases were disposed off in a protected burial pit near the post-mortem facility.

The same procedures were used for both baseline and endline carcase dissections. All personnel involved in the carcase dissections were blinded as to which treatment group/trial site a pig belonged and pigs were slaughtered in no particular order with respect to their groups or origin.

### Ethical considerations

Conduct of the study was authorized by the Director of Veterinary Services (DVS) of the Ministry of Livestock and Fisheries Development. The study protocol was approved by Research, Publications and Ethics committee of the College of Veterinary Medicine and Biomedical Sciences (CVMBS) of the Sokoine University of Agriculture, SUA (Reference number: SUA/CVMS/016/32). Permission to import and use the investigational materials (vaccine and OFZ) was granted by the then Tanzania Food and Drugs Authority (TFDA). Prior to study start, owners of the animals signed informed consent forms to authorize the use of their animals in the study and to agree to comply with all study protocol requirements. In case an owner could not read or write, a verbal consent was provided in the presence of a witness after the information was read and explained. The study was conducted as guided by the principles of good clinical practice. All regulatory requirements including applicable animal welfare regulations were strictly complied with and adhered to section 40 of the Tanzania’s Animal Welfare Act of 2008.

### Data analysis

Raw data was transcribed into pre-formatted excel spreadsheets and imported into R version 3.6.0 and GenStat version 19.1 for analysis. Baseline values for each of T1 and T2 groups were used as their respective baseline control values.

The prevalence of viable *T*. *solium* cysticerci was calculated with exact 95% confidence intervals. Fisher’s exact test was used to compare baseline and endline prevalence for each treatment group, between the endline prevalence of these groups and their respective controls and between baseline and endline values for the controls. Firth’s Bias-reduced Penalized Maximum Likelihood method, used for the analysis of binary outcomes for small samples, was used for each treatment group separately to model the main effects of stage of study (baseline/endline), treatment group (T1, T2, control) and the interaction effect between stage of study and treatment group.

Non-parametric random permutation t-tests were run on natural log-transformed viable cysticerci count data to make the same comparisons as above for T1. However, as endline T2 cysticerci count was zero, alternative 2-sided one sample t-tests were run for baseline and endline T2 control, to determine whether either were significantly different from zero. All comparisons were considered statistically significant at two-sided *p* <0.05.

## Results

### Sex, estimated age and management practices of treated pigs

During the entire intervention period, herds contained more females (>61%) than males. In the first round of interventions, mean age was 7.9 (± 6.5) months, ranging from 2 to 48 months and pig weight ranged from 3 to 147 kg with a mean of 24.3 kg (± 19.7). During the second round, mean age increased to 10.3 (± 6.4) months ranging from 2 to 52 months. Mean weight also increased to 29.9 kg (± 19.2) with a range of 4 to 151 kg. During the third round, the mean age was 10.9 (± 7.3) months and ranged from 2 to 60 months. Mean weight was 30.7 (± 18.7) with a range of 3 to 151 kg. It was noticeable that the herds were comprised of older pigs in the second and the third rounds compared to the first round of interventions.

### The intervention

The number of households visited and pigs treated during all intervention rounds are shown in Table 2. At the start of the intervention, a total of 1,359 pigs from 498 pig keeping households were enrolled. It was estimated that the enrolled households represented about 90% of all pig keeping households in the selected villages. At least 74% of pigs in the households satisfied the inclusion criteria and were enrolled. Majority of pigs (nearly 70%) which could not be enrolled in the selected households were piglets below the age of two months. In subsequent rounds there were changes in the numbers of pigs and households due to new enrolments and losses to follow-up, but overall, the number of treated pigs decreased at each round. During the entire intervention period, the total number of treatments was 3,309, an average of 1,103 per round. Out of those, 1,869 were treatments with OFZ alone while 1,440 were concurrent treatment with OFZ and vaccination with TSOL18.

**Table 2:** Number of households visited and of pigs treated throughout the intervention period in Mbeya Rural and Mbozi districts, southern highlands of Tanzania.

		First round			Second round			Third round		
		Mbeya	Mbozi	Total	Mbeya	Mbozi	Total	Mbeya	Mbozi	Total
**Households**	T1	130	112	242	111	120	231	84	104	188
	T2	124	132	256	79	102	181	62	76	138
	Total	254	244	498	190	222	412	146	180	326
**Pigs**	T1	399	295	694	294	361	655	276	244	520
	T2	294	371	665	257	175	432	125	218	343
	Total	693	666	1359	551	536	1087	401	462	863

T1, intervention with oxfendazole only; T2, concurrent intervention with TSOL18 and oxfendazole.

The number of pigs enrolled at each round, pigs left out because of not meeting inclusion criteria, pigs re-treated at subsequent rounds and losses to follow-up are shown in Table 3. Reasons for loss to follow-up included pig deaths, sales, slaughters and transfers to distant non-study villages. Others were failure to capture free roaming pigs and owners opting out of the study. African swine fever (ASF) was reported to be responsible for most cases of deaths. An outbreak of ASF occurred after the first intervention round in some of the study villages. Apart from causing pig deaths, the outbreak led to some farmers opting out of the study because they were misinformed and hence associated the deaths to the vaccination/treatment. About 2.8% of pigs which were lost to follow-up belonged to owners who opted out of the study. No adverse reactions were observed or reported as a result of vaccination/treatment. A single suspected case of an adverse event was observed during the third round of intervention where a young pig was seen to be disoriented soon after injection/drenching. However, the signs were short-lived and the case resolved itself without intervention, and it was assumed that other causes were responsible.

**Table 3:** Number of pigs enrolled at each round, pigs not meeting inclusion criteria, pigs re-treated at subsequent rounds and losses to follow-up at each round of intervention in both intervention groups in Mbeya Rural and Mbozi districts, southern highlands of Tanzania.

Intervention round	Number of enrolled pigs			Pigs not meeting inclusion criteria			Pigs re-vaccinated /re-treated			Losses to follow-up		
	**T1**	**T2**	**TOTAL**	**T1**	**T2**	**TOTAL**	**T1**	**T2**	**TOTAL**	**T1**	**T2**	**TOTAL**
1	694	665	1359	136	99	235	NA	NA	NA	NA	NA	NA
2	212	98	310	72	48	120	438	359	777	222	360	582
3	169	117	286	57	199	256	352	225	577	283	227	510
TOTALS	**1075**	**880**	**1955**	**265**	**346**	**611**	**790**	**584**	**1354**	505	587	**1092**

T1, intervention with oxfendazole only; T2, concurrent intervention with TSOL18 and oxfendazole; NA, not applicable.

At the end of the intervention, a total of 577 pigs (352 from T1 and 225 from T2) had received at least two treatments/vacinations, hence were eligible for selection for the endline carcase dissections. The number of treatments that pigs slaughtered for endline carcase dissections had received are shown in Table 4.

**Table 4:** Number of animals which were slaughtered an intervention with TSOL18 and oxfendazole in Mbeya Rural and Mbozi districts, southern highlands of Tanzania.

Intervention round	T1		T2		Totals	
	n	%	n	%	N	%
R2+R3	14	38.9	22	28.6	35	31
R1+R2+R3	20	55.5	51	66.2	72	63.7
R1+R3	2	5.6	4	5.2	6	5.3
TOTALS	36	100	77	100	113	100

N, number of pigs slaughtered; R1, R2, R3-first, second and third intervention rounds, respectively; T1, intervention with oxfendazole only; T2, cconcurrent intervention with TSOL18 and oxfendazole.

### Prevalence of viable *T*. *solium* cysticerci

The number of pigs slaughtered and prevalence of viable *T*. *solium* cysticerci at baseline and endline are summarized in Table 5. The baseline and endline prevalence for T1 were 25.5% (Confidence interval [CI]: 13.9, 40.3%) and 2.8% (CI: 0.1, 14.5%), respectively. For T2, prevalence were 12% (CI: 6.4, 20.0%) at baseline and 0% (CI: 0, 4.7%) at endline. The endline prevalence for the untreated pigs was 12.5% (CI: 0.3, 52.7%) for T1 and 5.7% (CI: 0.7, 19.2%) for T2. Fisher’s exact pairwise comparison showed that the change in prevalence of infection with viable *T*. *solium* cysticerci from baseline to endline in T1 (from 25.5% to 2.8%) was significant (*p =* 0.005). However, the difference between T1 and its control at endline (2.8% vs. 12.5%); and between controls at baseline and endline (25.5% vs. 12.5%) were not significant (*p =* 0.334 and *p =* 0.664, respectively). From Firth’s penalized ML estimations, the interaction effect between the stage of trial and treatment was not significant (*p =* 0.245) indicating that there was no significant effect of the intervention when taking into account prevalence in untreated pigs. The lack of statistical significance between the T1 and its control, in the change in positivity from baseline to endline (T1 = 22.8%, T1 control = 13%) may be partly due to the low numbers of control observations at endline. Similarly, the 95% exact confidence intervals for the T1 baseline and endline and its endline control are wide due to the lower than required sample size for T1 and a very small sample size (n = 8) for its control.

**Table 5:** The number of pigs slaughtered and prevalence of *Taenia solium* cysticercosis in study treatment groups and controls at baseline and endline in Mbeya Rural and Mbozi districts, southern highlands of Tanzania.

Stage of study		Study treatment group	
		T1	T2
Baseline	Examined (n)	47	100
	Infective (n)	12	12
	Prevalence % (95% CI^1^)	25.5 (13.9, 40.3)	12.0 (6.4, 20.0)
Endline (treatments)	Examined (n)	36	77
	Infective (n)	1	0
	Prevalence % (95% CI)	2.8 (0.1, 14.5)	0 (0, 4.7)
	Infected pigs^2^ (n)	3	0
	Prevalence % (95% CI)	8.3 (1.8, 22.5)	0 (0, 4.7)
Endline (controls)	Examined (n)	8	35
	Infected (n)	1	2
	Prevalence % (95% CI)	12.5 (0.3, 52.7)	5.7 (0.7, 19.2)

T1, intervention with oxfendazole only; T2, concurrent intervention with TSOL18 and oxfendazole.

1 95% Exact confidence intervals.

2 Two of the three infected pigs had non-viable cysticerci only.

For the T2 group, Fisher’s exact test for comparisons showed that there was a significant difference in prevalence of infection with viable *T*. *solium* cysticerci from baseline to endline (12% vs. 0%, *p* = 0.001). However, the difference between T2 and its control at endline (0% vs. 5.7%), and between controls at baseline and endline (12% vs. 5.7%) were not significant (*p* = 0.096 and *p* = 0.519, respectively). Using the Firth’s penalized ML method, there was some, yet not significant evidence of interaction between stage of trial and treatment (*p* = 0.076) with T2 showing a greater difference in prevalence of viable infections between baseline and endline (difference = 12%) than its control difference = 6.3%). The sample sizes for both T2 baseline and endline were close to the required sample size, resulting in smaller confidence intervals than those seen for T1. However, the T2 control remained with a wide 95% confidence interval (0.7–19.2%) given a relatively smaller sample size (n = 35).

### Counts of viable *T*. *solium* cysticerci

The number of cysticerci and percentage of viable cysticerci in different organs and muscle groups of pigs slaughtered at baseline and endline are shown in Table 6. In T1, a random permutation t-test on natural log-transformed (Log_e_) count data showed a significant difference between baseline and endline counts (*p =* 0.017, mean difference = 1.093, 95% CI = 0.217, 1.969). However, there was no difference between T1 and its control at endline (*p =* 0.852) and between controls at baseline and endline (*p =* 0.965).

**Table 6:** Total counts of *Taenia solium* cysticerci and percentage of viable cysticerci in different organs and muscle groups of pigs slaughtered at baseline and endline in Mbeya Rural and Mbozi districts, southern highlands of Tanzania.

	Baseline				Endline^1^					
Organ/muscle group	T1 (n = 12/47)		T2 (n = 12/100)		T1 (n = 1/36)		T1 control (n = 1/8)		T2 control (n = 2/35)	
	Total	%viable	Total	%viable	Total	%viable	Total	%viable	Total	%viable
Brain	471	100	183	100	98	100	37	100	0	0
Tongue	3,316	100	2,456	100	783	82.1	1,120	80.1	18	100
Masticatory muscles	7,331	100	1,642	100	130	90	242	49.6	23	100
Heart	1,245	100	319	98.1	351	64.4	2,005	95.1	27	96.3
Diaphragm	1,173	100	651	100	113	95.6	250	100	15	93.3
Forelimb	21,320	100	4,912	100	2,162	19.1	18,060	79.8	108	100
Remaining half carcase	13,768	100	19,720	100	588	7.1	27,996	85.7	224	99.1
Totals	48,624	100	29,883	100	4,225	39	49,710	83.7	415	99

T1, intervention with OFZ only; T2, concurrent intervention with TSOL18 and OFZ.

1 No pig from T2 had *T*. *solium* cysticerci at endline.

In T2, the random permutation t-test showed no difference between T2 at baseline and its control at endline (*p* = 0.404). The 2–sided one sample t-test results for the T2 baseline and T2 endline control showed that T2 baseline counts differed significantly from zero (*p* = 0.004, mean = 0.5463, 95% CI = 0.179, 0.913) as opposed to T2 endline control which showed no evidence of difference (*p* = 0.160). Hence, there was evidence that the cysticerci count was reduced between baseline and endline but not that there is a difference at endline between T2 and its control.

## Discussion

This field study evaluated the effectiveness of concurrent administration of TSOL18 vaccine and OFZ; and OFZ alone, provided at least two times at an interval of four months to pigs exposed to natural infection of *T*. *solium* in an endemic area in Tanzania. The results showed that concurrent administration with TSOL18 and OFZ cleared *T*. *solium* infection in the assessed pigs, when measured 2 to 3.5 months after the last intervention. However, relative to the untreated control group assessed contemporaneously at endline, the difference was not statistically significant; although there was some evidence of T2 showing a greater difference in prevalence between baseline and endline (12.0%) than its control (6.3%).

The average age of the treated pigs at slaughter was 17 months. The ages of the untreated pigs were not recorded, although it has previously been reported that in the area pigs are usually slaughtered when they are around 12 months [26]. Hence we can speculate that, overall, the treated pigs were older than untreated pigs and this might have presented an uncontrolled bias when comparing treated and untreated pigs at endline.

The 100% efficacy of the use of both TSOL18 and OFZ in treated pigs is consistent with the previously published results from studies in Cameroon [27] and Nepal [28]. In the study in Cameroon, two immunizations with TSOL18 were provided four weeks apart, with OFZ provided concurrently at the second immunization. A booster immunization followed three months after the second immunization. The intervention was 100% efficacious as no cysticerci were found in any of the vaccinated pigs. Similarly, in a recent study in Nepal, an intervention with 3–monthly pig vaccination with TSOL18 and treatment with OFZ cleared infection amongst vaccinated pigs.

This study has also shown that treatment with OFZ alone resulted in a statistically significant difference in prevalence of infection with (and counts of) viable cysticerci from baseline to endline. However, as for the concurrent administration with TSOL18 and OFZ, the prevalence (and cysticerci counts) was not significantly different when taking into account untreated control pigs assessed contemporaneously at endline, 2 to 3.5 months after the last intervention. In T1, infection remained at the end of the trial, as three pigs were infected at endline, two of them with non–viable cysticerci only. However, this was an expected finding as OFZ kills cysticerci within days of treatment but residues of cysticerci persist for months [29].

Although no statistically significant differences in prevalence and counts of viable cysticerci were observed in the treated groups relative to their untreated controls, it is important to consider that the chances for accurately determining the differences were compromised by a lack of power. This was particularly the case for the T1 untreated group, due to the small sample size (n = 8) and wide 95% exact confidence interval (0.3%– 52.7%), whereas the confidence interval for T2 untreated group was relatively narrow (0.7%– 19.2%, n = 35) and some evidence of a reduction in prevalence relative to untreated controls was seen (*p* = 0.076).

The carcase dissections in this trial were performed on average 70 days after the final treatments. This provided only a relatively short period of time for the animals to be exposed to infection and develop detectable viable cysts. Logistical challenges prevented the pig necropsies being undertaken at a later time. This limitation may have affected particularly the outcome of the group receiving oxfendazole treatment alone because the majority of them would have had no immunity to an exposure to the parasite. Hence more cases of viable infection in this group may have been found had the necropsies been undertaken at the time the next oxfendazole treatment would have been due, that is, if the oxfendazole treatments were to be continued as part of an on-going control program.

This study has also shown that even after the interventions, there were infected pigs among the untreated pigs in both intervention areas, indicating that transmission of *T*. *solium* was still ongoing in the untreated pig populations in the areas. This is most likely because no intervention on humans was included, hence environmental contamination with the *T*. *solium* cysticerci continued and so untreated pigs remained at risk of infection. In addition, these interventions were carried for a relatively short duration and did not include all pigs in the study areas. Theoretically, it is safe to assume, at least for the T2 group, that if the intervention on pigs only would be continued for a duration beyond the acknowledged life span of the tapeworm (2–3 years), the transmission would probably be interrupted. Hence, to have an effect on taeniosis (and eventually on NCC) effective interventions on pigs only need to be carried out for a period of at least 2–3 years [30]. However, this will very much depend on the extent of environmental contamination with *Taenia* eggs and for how long the eggs can survive in the environment. However, theoretical models indicate that interventions targeting both human and porcine hosts are more likely to succeed than intervention applied on a single host [31–34]. So, although the interventions assessed in this trial provide some prospects for the control of porcine cysticercosis, a larger and rapid impact on the transmission of the disease, particularly among humans, would be achieved by also strategically treating the human population with an anthelmintic, and also addressing environmental contamination with *Taenia* eggs.

It shall further be noted that in this study, the minimal number of pigs for carcase dissection was only achieved for T2 at baseline leading to lower power for the study to detect the anticipated reduction in prevalence. The smaller than required sample sizes, especially at endline were partly due to occurrence of ASF in the study areas which led to the reduction of the number of pigs which could be available for slaughter. However, greater reduction was observed from higher initial prevalence for T1 and therefore the non–parametric tests were able to show statistically significant reductions between baseline and endline for both groups. Sample sizes were not based on cysticerci counts but for this parameter it was also possible to show a significant reduction for T1 and T2.

The drawback entailing the use of TSOL18 is the necessity of maintaining the cold chain for the vaccine to be effective. This may be a challenge in many endemic areas where the supply of electricity is not reliable. As for the use of OFZ, the necessity of observing the 3 weeks withholding period may impose a burden on some farmers. Most farmers keep pigs as a mobile bank from which they can draw quick cash whenever an urgent need arises, so compliance with the withdrawal period may be difficult to observe and to monitor. The inability of OFZ to kill all brain cysticerci is another drawback although consumption of undercooked pig brain has been reported to be uncommon in endemic areas [35].

This study has employed carcase dissection as a diagnostic method to measure intervention effectiveness. The method, despite being currently regarded as the gold standard for the diagnosis of PC, has a disadvantage of being costly and labour intensive. Further, it necessitates removal of a certain proportion of the pig population and this might affect transmission dynamics and pork value chains. Hence, feasibility of carcase dissection in monitoring and/or evaluating intervention programmes may be compromised [36]. Therefore, there is an urgent need for the development of an affordable, field-friendly ante-mortem diagnostic test with good sensitivity and specificity.

A limitation of this study that should be acknowledged was the lack of a control group from the beginning of the study. We could not be certain that the control pigs had been in areas and exposed to the same environmental factors all along the trial period, as the treated pigs. Therefore, it could not unequivocally be established if other elements, such as changes in disease occurrence, changed at the same time as the intervention was implemented in the study areas.

In conclusion, a difference in prevalence and cysticerci counts of *T*. *solium* cysticerci in smallholder pigs kept under field conditions and exposed to natural infections of *T*. *solium* in endemic areas of Tanzania was observed in sites after some pigs were treated with at least two treatments of OFZ alone or the concurrent administration of TSOL18 vaccine and OFZ. Although the differences between baseline and endline prevalence and cysticerci counts were significant, they could not be definitively attributed to the effect of either intervention because of lower than required sample sizes particularly for the untreated pigs. Further investigation, including larger sample sizes for both treated and untreated pigs, would be required to determine whether the observed differences could be attributed to either intervention. In addition, although a recent study has shown no significant relationship between age of animals and proportion that was infected *with T*. *solium* [28], future studies must consider reducing the age-range for inclusion of pigs to remove any possible age-related bias. Also, assessment of cost-effectiveness of both interventions will help in making informed decisions. This study has also shown that infected pigs remained among pigs treated with OFZ alone whereas concurrent treatment with both TSOL18 and OFZ cleared infection among treated pigs. Presence of infective pigs after either intervention showed that transmission remained in the areas. A One Health approach including treatment of humans, and incorporating the environment will have a bigger and sustainable impact.
